# Physicochemical and Antibacterial Properties of Chitosan Extracted from Waste Shrimp Shells

**DOI:** 10.1155/2016/5127515

**Published:** 2016-07-13

**Authors:** José Carlos Vilar Junior, Daylin Rubio Ribeaux, Carlos Alberto Alves da Silva, Galba Maria De Campos-Takaki

**Affiliations:** ^1^Northeast Network for Biotechnology, Federal Rural University of Pernambuco, 52171-900 Recife, PE, Brazil; ^2^Nucleus of Research in Environmental Sciences and Biotechnology, Catholic University of Pernambuco, 50050-590 Recife, PE, Brazil; ^3^Doctorate Program in Biological Sciences, Federal University of Pernambuco, 50670-420 Recife, PE, Brazil

## Abstract

This research aims to study the production of chitosan from shrimp shell (*Litopenaeus vannamei*) of waste origin using two chemical methodologies involving demineralization, deproteinization, and the degree of deacetylation. The evaluation of the quality of chitosan from waste shrimp shells includes parameters for the yield, physical chemistry characteristics by infrared spectroscopy (FT-IR), the degree of deacetylation, and antibacterial activity. The results showed (by Method 1) extraction yields for chitin of 33% and for chitosan of 49% and a 76% degree of deacetylation. Chitosan obtained by Method 2 was more efficient: chitin (36%) and chitosan (63%), with a high degree of deacetylation (81.7%). The antibacterial activity was tested against Gram-negative bacteria (*Stenotrophomonas maltophilia* and* Enterobacter cloacae*) and Gram-positive* Bacillus subtilis* and the Minimum Inhibitory Concentrations (MIC) and the Minimum Bactericidal Concentration (MBC) were determined. Method 2 showed that extracted chitosan has good antimicrobial potential against Gram-positive and Gram-negative bacteria and that the process is viable.

## 1. Introduction

Chitin is the most abundant organic substance in nature after cellulose. This biopolymer has a highly linear structure and is insoluble in water. It dissolves quickly in concentrated acids and in some fluoroalcohols yet its reactivity is low. Other relevant properties of this biopolymer are its high molecular weight and porous structure which favors high water absorption [1, 2]. On the other hand, chitosan is obtained from this biopolymer and this has a structural function. These associations act as a matrix that interacts with other constituents such as phenolic tannins in insects and minerals in the carapaces of crustaceans [3, 4].

Deacetylation is the nonenzymatic process whereby chitosan is obtained by removing R-NHCOCH_3_ residue and treating it with strong alkali at high temperatures. When the degree of deacetylation is greater than 50%, the biopolymer becomes soluble in acidic aqueous solutions and behaves as a cationic polyelectrolyte due to the protonation of amine groups in the presence of H^+^ ions [5, 6]. Other ways to obtain chitosan are by using enzymatic processes. However, they are not used on an industrial scale owing to the high commercial cost of enzymes (deacetylases) and their low productivity, while nonenzymatic chemical processes are widely used because the cost of doing so is low and the processes are efficient [7, 8].

The development of new applications for chitosan is strongly based on the fact that this polymer can be obtained from renewable sources such as fisheries; it is nontoxic, nonallergenic, biodegradable, and present in antimicrobial activity [9].

Studies with planktonic crustaceans such as* Daphnia longispina* resting eggs indicate that these crustaceans can be exploited as a source of chitin due to their high chitin content (23~25%) [10].* Leptinotarsa decemlineata*, also known as the Colorado potato beetle, is a major pest of potato crops. The adult and larva have 20% and 7% of chitin content, respectively. However, the chitin from adult Colorado potato beetles had a more stable structure than that from the larvae. Investigation has indicated that the adult potato beetle is more appropriate as a chitin source, both because of its chitin and chitosan content and because of its antimicrobial and antioxidant activities [11].

Kaya et al. [12] conducted studies on potential sources of chitin in the Orthoptera order of insects* Calliptamus barbarus* and found this to be 20.5 ± 0.7% and 16.5 ± 0.7% for* Oedaleus decorus*, the yield of chitosan being 74–76%, with a deacetylation degree of 70–75%. The insects showed potential as alternative sources of chitin and chitosan on account of their antimicrobial and antioxidant properties for the food/animal feed industry.

Among the most common applications are their uses as complexing material metal ions, such as edible coatings with antifungal and bactericidal action [13–15] and as a basic element for making controlled drug delivery matrices [16].

Thus, the objective of this study was to investigate the efficiency of different methodologies to obtain chitosan from the waste of* Litopenaeus vannamei* shrimps since this raw material comes from renewable resources and it is economically viable to produce high-value added compounds from it.

## 2. Materials and Methods

### 2.1. Preparation of Raw Material

 Shrimp residues of the species named as* Litopenaeus vannamei* were washed in running water and a 2.5% hypochlorite solution. Thereafter, they were dried at room temperature and then crushed and passed through a 16-mesh knit.

### 2.2. Microorganisms

The bacteria* Stenotrophomonas maltophilia* (UCP-1600),* S. maltophilia* (UCP-1601),* Bacillus subtilis* (UCP-1002), and* Enterobacter cloacae* (UCP-1603) were kindly supplied from the Culture Collection UCP (Universidade Católica de Pernambuco), Recife, PE, Brazil. Culture Collection is registered in the WFCC (World Federation Culture for Collection). These microorganisms were used in the tests of evaluation of Minimum Inhibitory Concentration (MIC) and the Minimum Bactericidal Concentration (MBC). The microorganisms were maintained at 25°C in nutrient agar medium (peptone 0.5%, beef extract 0.3%, NaCl 0.5%, agar 1.5%, and distilled water, and pH is adjusted to neutral 7.4).

### 2.3. Chitin and Chitosan Extraction

The extraction of chitin and chitosan was performed according to the methods described by Zamani et al. [17] (Method 1) and Arantes [18] (Method 2). In order to eliminate the proteins of the residue, NaOH solutions 0.5 M (30 : 1 v/m, 90°C, 2 h) and 0.3 M (10 : 1 v/m, 80°C, 1 h, under agitation), respectively, were used. Then, the alkali-insoluble fraction (IFA) was separated by centrifugation at 4000·*g* for 15 minutes and/or by vacuum filtration. Subsequently, to demineralize the precipitate obtained, 10% acetic acid (100 : 1 v/m, 60°C, 6 h) and 0.55 M hydrochloric acid (10 : 1 v/m, room temperature, 1,5 hours) were used. To obtain purified chitosan, treatments with 1% sulfuric acid (121°C/20 min) and 50% NaOH (100°C, 10 h) were performed.

### 2.4. Spectroscopy to Infrared Ray (IR)

Two milligrams of chitin and chitosan samples was dried overnight at 60°C under reduced pressure; then, this was homogenized with 100 mg of KBr. Discs with the prepared KBr were dried for 24 h at 110°C under reduced pressure. The chitin and chitosan samples from shrimp shell (*Litopenaeus vannamei*) waste were analyzed at 4000–625 cm^−1^ using an infrared ray Fourier Transform Spectrometer (FT-IR, BRUKER Mod. IFS). A KBr disc was used as reference. To determine the maximum absorption intensity of bands, the baseline was used.

### 2.5. Determination of the Deacetylation Degree (DD%)

The degree of acetylation and deacetylation of chitosan was determined using an infrared ray spectroscopy, IR 22, applying the band *A*_1655_/*A*_3450_ which was calculated as per [19]

(1) $$\begin{array}{c} AD\left(\;\%\;\right)=\left(\;\frac{A_{1655}}{A_{3450}}\;\right)\times \frac{100}{\text{1,33}}. \end{array}$$

### 2.6. Evaluation of Minimum Inhibitory Concentration (MIC)

To evaluate the Minimum Inhibitory Concentration (MIC), the serial dilution technique was used with the tested microorganisms, in accordance with Qi et al. [20]. An initial chitosan solution was prepared at 0.5% in 1% acetic acid and pH = 5.0. Then, serial dilutions were performed of 1 : 1 to 1 : 512 and decreasing concentrations ranging from 0.00005% to 0.25%. For each microorganism, a standard bacterial suspension was prepared in sterile saline solution and compared to the 0.5 McFarland scale tube. 10 *μ*L bacterial suspension was transferred to each one in the series of tubes and incubated at 37°C for 24 hours.

### 2.7. Evaluation of Minimum Bactericidal Concentration (MBC)

For the evaluation of Minimum Bactericidal Concentration (MBC), a qualitative technique was used according to the method of Qi et al. [20]. The series of chitosan solutions, which determined the MIC, were used to evaluate MBC. From the reading of the MIC, the tubes that showed no visible turbidity had 10 *μ*L plated on blood agar, pH 7.0, and were incubated for 24 h at 37°C, and observations were made on whether or not the colonies of microorganisms grew.

## 3. Results and Discussion

According to elementary studies and analyses of different crustaceans (shrimp, lobster, and squid), there was great variability of this composition when chitin amounts were varied for squid of approximately 1.8%, pink shrimp 22% (under study), and lobster 36% [21]. Hence, there is a need to develop efficient demineralization and deproteinization processes to remove mineral content (20–30%) and protein content of approximately 40% in order to obtain chitin that is free of inorganic and protein content. This study showed that different concentrations of NaOH and demineralization with hydrochloric acid and acetic acid influenced the yield of the extraction process used to obtain chitin and chitosan. Similarly, it was proved that the methods used also had an effect on the degree of deacetylation (Table 1). To confirm that the biopolymer was chitosan, the product obtained with the commercial chitosan Sigma (Sigma Aldrich Corp., St. Louis, MO, USA) was characterized and compared by infrared spectrometry.

The residual mass from shrimp exoskeleton after demineralization and deproteinization processes showed well preserved chitin structure as described by Stamford et al. [22]. This was higher than the values obtained by Tenuta Filho and Zucas [23], with 14% of chitin pink shrimp waste (*Penaeus brasiliensis*) and by Beaney et al. [24] with 10% yield of biopolymer from* Nephrops norvegicus*.

Kaya et al. [25] found that the chitin content of bat guano species* Rhinolophus hipposideros* collected from Karacamal Cave was 28%. It was noted the chitosan productivity corresponding to 79% from isolate chitin is superior to our results from* L. vannamei* using two different methodologies. However, the chitosan content from* L. vannamei* using two methods showed better results.

A new chitin source was described by Kaya et al. [26] using* Gammarus argaeus* Crustacea. The results showed the isolation of alpha chitin and were confirmed by infrared spectroscopy, thermogravimetric analysis (TGA), X-ray diffraction (XRD), and scanning electron microscopy (SEM) techniques.

More recently, the production of a new morphology of chitin from the wings of* Periplaneta americana* has been studied by Kaya and Baran [27]. They showed the surface of the chitin has oval nanopores (230–510 nm) without nanofibers. The chitin surface had a pore in the center and six or seven other pores distributed around these, corresponding to structures similar to cell walls. Alternatively, studies with chitin content of the structure of the exoskeleton of seven species from grasshopper of the four genera were carried out [28]. The contents of chitin varied between 5.3% and 8.9% and had a low molecular weight (between 5.2 and 6.8 kDa). A large amount of waste is formed from invasive and harmful species that have been killed by the use of insecticides, and the authors suggest that these be collected and evaluated as an alternative chitin source.

Some parameters in the deacetylation reaction are cited as fundamental factors on the end characteristics of chitosan [29]. Tsaih and Chen studied the influence of temperature and processing time on polymer chitosan characteristics and found that both have a significant effect on the deacetylation degree and molecular weight [30].

The results obtained also showed a higher yield than that found for the chitin extracted from shrimp* Penaeus brasiliensis* [31], which was 5.3% and 2.5% of chitosan. Santos et al. [32] showed a lower percentage with 5.9 and 5.06% of chitin and chitosan, respectively. Thus, the maximum chitosan obtained from chitin deacetylation (57.5%) was similar to the reported value for the extraction from the polymer using the shrimp* Macrobrachium rosenbergii* (~65%). However, the results obtained by Battisti and Campana-Filho showed that 80% of chitin was transformed into chitosan [33].

**Figure 1:**
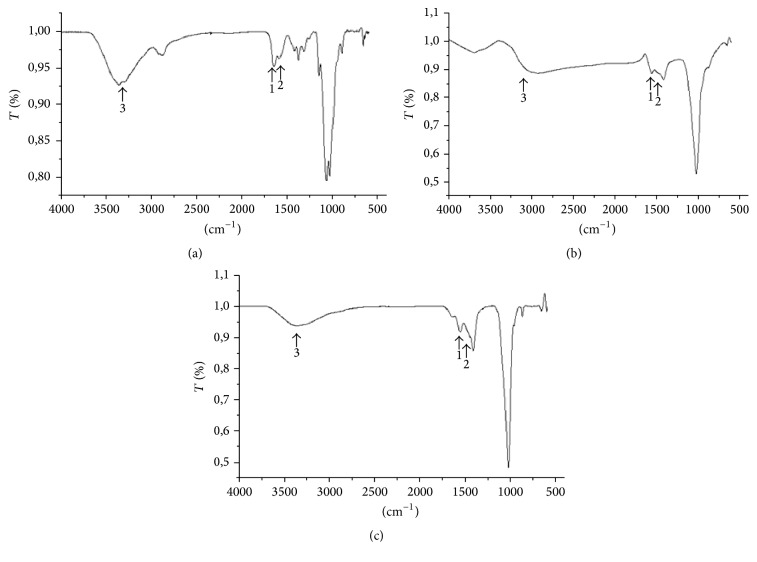
Infrared absorption spectra: (a) commercial chitosan (Sigma Aldrich Corp., St. Louis, MO, USA); (b) chitosan obtained by Method 1 [17]; (c) chitosan obtained by Method 2 [18]. The peaks in chitosan were indicated as 1 = (∼1650 cm^−1^) acetylated residues (NHCOCH_3_); 2 = (∼1590 cm^−1^) NH_2_ groups present in the deacetylate residues; and 3 = (3440 cm^−1^) the vibration of the OH molecule.

The spectrophotometric analysis of commercial chitosan (Figure 1(a)) and the chitosan obtained by the methods used (Figures 1(b) and 1(c)) enabled the bands to be characterized as follows: Peak 1 (~1650 cm^−1^) corresponded to acetylated residues (NHCOCH_3_) of chitosan; Peak 2 (~1590 cm^−1^) identified the NH_2_ groups present in the deacetylate residues; and Peak 3 (3440 cm^−1^) corresponded to the vibration of the OH molecule [34]. Analysis by FT-IR estimated the amount of free amine groups present in the molecule of chitosan obtained from the two methodologies, namely, 76% and 81.7%, respectively (Table 2). However, the higher deacetylation degree of chitosan is generally controlled by processing the native polymer with alkali and increasing time and temperature [30]. These values are consistent with commercial chitosan, obtained from crustaceans, since this reaches between 75 and 90% deacetylation in industrial processing.

In a study proposing a simple and efficient method of deacetylation of chitosan using acetate of 1-butyl-3-methylimidazole, as the reaction catalyst, DD% = 86 was obtained, a value similar to that found in our study in the best condition for producing the biopolymer (DD = 81.7%) [35].

Santos et al. [32] determined the degree of deacetylation of chitosan obtained from the shrimp “Saburica” (*Macrobrachium jelskii*), which was approximately DD 76%, using linear potentiometric titration. The results using Fourier transform infrared spectroscopy obtained in this study are in agreement with the data in the literature, which may vary from 50 to 92.3% [36, 37].

Hennig [31] analyzed obtaining chitosan from* Penaeus brasiliensis* and obtained a degree of deacetylation (DD%) of 87%. This value was similar to that reported in the literature for Weska et al. [38] and to those obtained in the best condition.

Furthermore, it was shown that the chitosan produced has characteristics comparable to commercial chitosan, the degree of deacetylation ranging between 70 and 95% [36, 39].

Recently, Kaya et al. [40] undertook studies on chitin obtained from Insecta (*Melolontha melolontha*) and Crustacea (*Oniscus asellus*) and compared their physical and chemical properties. The results showed chitin content for dry weights of* M. melolontha* and* O. asellus* corresponding to 13-14% and 6-7%, respectively. The results observed that chitin nanofibers of* O. asellus* adhered to each other; nanofibers of* M. melolontha* were nonadherent and were considered the more attractive chitin source.

Studies were carried out with* Fomitopsis pinicola*, a medicinal fungus in Asia, and found 30.11% of chitin and yield of 71.75% of chitosan from the dry weight. The chitin showed acetylation of 72.5% and deacetylation of chitosan was 73.1%, and the maximum chitin temperature of degradation was 341°C [41]. Results clearly revealed a significant deacetylation degree of chitosan from waste shrimp shell* Litopenaeus vannamei* using two methodologies in comparison with deacetylation degree of chitosan determined to* F. pinicola* [41].

Fourier transform infrared spectroscopy (FT-IR), elemental analysis (EA), thermogravimetric analysis (TGA), X-ray diffractometry (XRD), and scanning electron microscopy (SEM) were used to investigate chitin structure isolated from both sexes of four grasshopper species, and it was observed that the amount of chitin was greater in males than females [42].

The physicochemical properties of chitin are investigated and related to taxonomy. The results showed those chitins properties are affected in different parts of the body (head, thorax, abdomen, legs, and wings) of the honey bee related to the extraction method. Physical and chemical properties form a parameter involved with taxonomy, and the chitin extracts from different parts of the body are different [43].

The influence of chitosan extracted by the methods proposed on inhibiting the growth of* Stenothrophomonas maltophilia* (UCP-1600/UCP-1601),* Enterobacter cloacae* (UCP-1603), and* Bacillus subtilis* (UCP-1602) is shown in Table 2. The actual mechanism of inhibition is not fully understood. However, the more acceptable hypothesis is related to a change in the permeability of the cell due to interactions between the biopolymer chitosan, when it is positively charged (pH below 6.5), and the cell membrane of microorganisms when negatively charged [44].

In the present study, the results demonstrated that MIC and MBC were more significant in Gram-negative bacteria when compared with Gram-positive ones but were effective in both cases. These results are in agreement with reports in the literature that have documented antimicrobial activity of chitosan against a large number of microorganisms, the MIC ranging between 0.1% and 1% [45, 46]. Thus, the efficiency of the chemical-physical characteristics is also related, as well as species or strains of bacteria in the same study [47, 48].

Wang [49] demonstrated that, for bactericidal action of chitosan on* E. coli, *solutions with concentrations between 0.5 and 1% at 48 hours had to be used, and to obtain the same effect at 24 h, higher concentrations of 1% chitosan were prepared. In addition, Tsai and Su [50] demonstrated that solutions of chitin and chitosan of high molecular weight and a high degree of deacetylation had a lethal effect on* E. coli* and* Shigella dysenteriae* when concentrations between 50 and 500 ppm were used. According to Chung et al. [47], the hydrophilicity of the cell wall and the negatively charged cell surface was greater in Gram-negative bacteria in relation to Gram-positive bacteria. In addition, the distribution of negative charges on their cell surfaces was very different when compared with Gram-positive bacteria, thus supporting the results found in this study.

In a study conducted to evaluate the bactericidal activity of glucose-chitosan complex of* E. coli*,* Pseudomonas*,* Staphylococcus aureus, and Bacillus cereus,* it was determined that the Minimum Inhibitory Concentration of chitosan was around 0.05%, these results being the same as those found in this study [51]. Moreover, on using the chitosan extracted from* Rhizopus arrhizus* and* Cunninghamella elegans* to evaluate the MIC and MBC on* Listeria monocytogenes*,* Staphylococcus aureus*,* Pseudomonas aeruginosa*,* Salmonella enterica*,* Escherichia coli,* and* Yersinia enterocolitica*, it was observed that the MIC values ranged from 200 *μ*g·mL^−1^ for* E. coli* to 500 *μ*g·mL^−1^ of* L. monocytogenes*. However, for the MBC, the results were between 400 *μ*g and 1000 *μ*g/mL^−1^, respectively [52]. The values determined in this study corroborate the results shown in Table 2. Thus, in this research study, the effect of the chitosan obtained by the proposed methods was proved to be effective as an antimicrobial agent on the microorganisms tested. Hence, the efficiency of applying this biopolymer in the therapeutic area was confirmed.

## 4. Conclusion

The previous results recommend Method 2 for the chemical reaction as it offers a clean, cheap, and convenient method for extracting chitosan from chitin extracted from shrimp wastes. Within the results in this work, the conclusion was reached that shrimp wastes are an excellent source for chitin. The yields and acetylation degree of chitosan decreased the concentration of NaOH solution, the temperature, and the length of treatment. The chitosan obtained showed the highest degree of deacetylation. Different chitosans were tested and markedly inhibited the growth of most bacteria tested; however, the inhibitory effects differed depending on the types of chitosan and the bacteria tested, there being greater antimicrobial activity against Gram-positive bacteria than against Gram-negative bacteria.

## Figures and Tables

**Table 1:** Chitin and chitosan copolymers obtained by the proposed methodologies.

Method	Shell waste (g)	Copolymers		Degree of deacetylation (%)
		Chitin (%)	Chitosan (%)	
Method 1 Zamani et al. [17]	10	33	49	76.0
Method 2 Arantes [18]	10	36	63	81.7

**Table 2:** Minimum Inhibitory Concentration (MIC) and Minimum Bactericidal Concentration (MBC) of chitosan by Zamani et al. [17] and Arantes [18] on Gram-positive and Gram-negative bacteria.

Microorganisms	Method 1		Method 2	
	MIC/*μ*g·mL^−1^	MBC/*μ*g·mL^−1^	MIC/*μ*g·mL^−1^	MBC/*μ*g·mL^−1^
*S. maltophilia* (UCP-1600)	156	312	312	312
*S. maltophilia* (UCP-1601)	78	156	78	156
*B. subtilis* (UCP-1002)	625	1250	625	625
*E. cloacae* (UCP-1603)	78	156	156	156
